# Sushi-Repeat-Containing Protein X-Linked 2: A Potential Therapeutic Target for Inflammation and Cancer Therapy

**DOI:** 10.1155/2022/2931214

**Published:** 2022-07-28

**Authors:** Jinhua Chen, Zhenhua Yin, Wenping Song, Baoxia He, Wenzhou Zhang

**Affiliations:** ^1^The Affiliated Cancer Hospital of Zhengzhou University & Henan Cancer Hospital, Henan Engineering Research Center for Tumor Precision Medicine and Comprehensive Evaluation, Henan Provincial Key Laboratory of Anticancer Drug Research, Zhengzhou 450008, China; ^2^Comprehensive Utilization of Edible and Medicinal Plant Resources Engineering Technology Research Center, Zhengzhou Key Laboratory of Synthetic Biology of Natural Products, Henan Joint International Research Laboratory of Drug Discovery of Small Molecules, Huanghe Science and Technology College, Zhengzhou 450063, China

## Abstract

Accumulating evidence has showed that sushi-repeat-containing protein X-linked 2 (SRPX2) is an abnormal expression in a variety of cancers and involved in cancer carcinogenesis, chemosensitivity, and prognosis, which mainly promote cancer cell metastasis, invasion, and migration by regulating the uPAR/integrins/FAK signaling pathway, epithelial-mesenchymal transition (EMT), angiogenesis, and glycosylation. Inflammation has been regarded as a key role in regulating cancer initiation, progression, EMT, and therapeutics. Furthermore, SRPX2 exhibited excellent antifibrosis effect *via* the TGF*β*R1/SMAD3/SRPX2/AP1/SMAD7 signaling pathway. Therefore, this review provides compelling evidence that SRPX2 might be a therapeutic target for inflammation and cancer-related inflammation for future cancer therapeutics.

## 1. Introduction

Sushi-repeat-containing protein X-linked 2 (SRPX2) is first found in 1999 by Kurosawa et al. that it is a downstream molecule of the *E2A-HLF* fusion gene in t (17;19)-positive leukemia cells. It is also called as sushi-repeat protein upregulated in leukemia (SRPUL) [1]. There are five genes in this family, including *SRPX2*, *SRPX* (*sushi-repeat-containing protein*, *X-linked*), *SVEP1* (*selectin-like protein*), *SELE* (*selectin E precursor*), and *SELP* (*selectin P precursor*) [2]. An increasing number of studies showed that SRPX2 is identified as a ligand for the urokinase plasminogen activator surface receptor (uPAR, CD87) [3]. As a crucial component of the extracellular plasminogen proteolysis system, uPAR is closely correlated with cancer invasion and metastasis, which is a transmembrane glycoprotein containing three distinct domains (D1, D2, and D3) [4, 5]. SRPX2 interacts with the D1 and D2-D3 extracellular domains of uPAR to enhance cancer invasion and metastasis by regulating multiple downstream signaling pathways, including the integrins/FAK pathway, the MAPK pathway, and the PI3K/Akt pathway [6, 7]. Roll et al. also found that transcription factor FOXP2, mutations of which caused severe speech and language disorders, could repress the interaction of SRPX2 and uPAR by directly binding to the promoter regions of SRPX2 (SRP1 and SRP2) and uPAR (UP2 and UP6) [8–10]. In addition, SRPX2 is regulated by its upstream molecules including TGF-*β*1, NFATC3, MAN1 (LEM) domain containing 1 (LEMD1), miR-192, miR-215, and miR-149 [11–14].

SRPX2 is an abnormal expression in a variety of cancers, such as pancreatic cancer, colorectal cancer, and gastric cancer [15–17]. Its overexpression in cancer has showed to relate with chemosensitivity, including cisplatin, nedaplatin, and temozolomide [14, 18]. Moreover, it might be a prognostic biomarker in cancer [17, 19]. Accumulating evidence has showed that inflammatory signaling pathways are closely involved in tumorigenesis, therapy resistance, and metastasis [20–22]. SRPX2 also exhibited excellent antifibrosis effect *via* TGF*β*R1/SMAD3/SRPX2/AP1/SMAD7-positive feedback loop, indicating that SRPX2 might be a therapeutic target for inflammation and cancer-related inflammation for future cancer therapeutics [11]. Therefore, the purpose of this review is to consider and analyze the role of SRPX2 in inflammation and carcinogenesis to provide insight into potential shared mechanisms.

## 2. Structure and Expression

**Figure 1:**
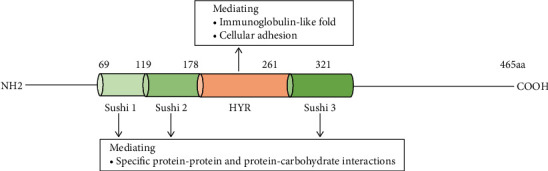
Schematic structure of SRPX2 protein. Sushi: sushi domain; sushi 1: 69-119AA; sushi 2: 120-178AA; sushi 3: 262-321AA; HYR: hyaline domain, 177-261AA; AA: amino acid.

On chromosome Xq22.1, the *SRPX2* gene encodes a secreted extracellular matrix protein SRPX2 (GenBank NP_055282), which was identified as a chondroitin sulfate proteoglycan later [23]. SRPX2 protein consists of 465 amino acids containing three consensus sushi domains and one hyaline (HYR) domain (Figure 1) [3]. A sushi domain, also known as a complement control protein (CCP) module or a short consensus repeat, mostly exists in proteins of the complement system and other diverse proteins including the selectin family. It consists of ~60 amino acids including four invariant cysteine residues forming two disulfide-bridges (I-III and II-IV), a highly conserved tryptophan, and conserved glycine, proline, and hydrophobic residues [24, 25]. Studies demonstrated that it was involved in the interactions of specific protein and protein or carbohydrate, such as interacting with hepatocyte growth factor (HGF) and uPAR [23]. Moreover, some eukaryotic proteins also have HYR domains in their structures, often relating with sushi domain. It is probably related to the immunoglobulin-like fold and involved in cellular adhesion [26].

**Figure 2:**
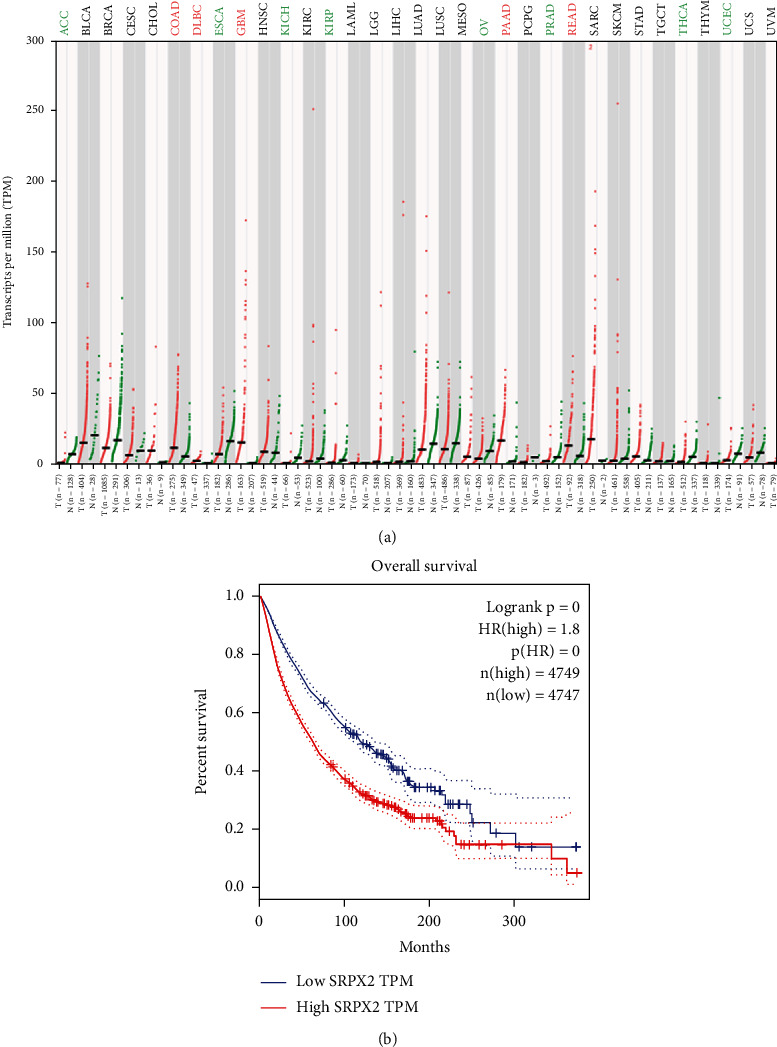
mRNA expression of *SRPX2* and survival analysis from the TCGA and GTEx database. (a) mRNA expression of *SRPX2* in 29 types of tumors. Green font represents that there is a lower expression of *SRPX2* in tumor tissue than that of the appropriate normal tissue. Red font represents that there is a higher expression in tumor tissue than that of the appropriate normal tissue. Black font represents that there is no significant difference in tumor tissue and the appropriate normal tissue. (b) The overall survival time of the SRPX2 high expression group (*n* = 4749) and the SRPX2 low expression group (*n* = 4747). ACC: adrenocortical carcinoma; BLCA: bladder urothelial carcinoma; BRCA: breast invasive carcinoma; CESC: cervical squamous cell carcinoma and endocervical adenocarcinoma; CHOL: cholangiocarcinoma; COAD: colon adenocarcinoma; DLBC: lymphoid neoplasm diffuse large B cell lymphoma; ESCA: esophageal carcinoma; GBM: glioblastoma multiforme; HNSC: head and neck squamous cell carcinoma; KICH: kidney chromophobe; KIRC: kidney renal clear cell carcinoma; KIRP: kidney renal papillary cell carcinoma; LAML: acute myeloid leukemia; LGG: brain lower-grade glioma; LIHC: liver hepatocellular carcinoma; LUAD: lung adenocarcinoma; LUSC: lung squamous cell carcinoma; MESO: mesothelioma; OV: ovarian serous cystadenocarcinoma; PAAD: pancreatic adenocarcinoma; PCPG: pheochromocytoma and paraganglioma; PRAD: prostate adenocarcinoma; READ: rectum adenocarcinoma; SARC: sarcoma; SKCM: skin cutaneous melanoma; STAD: stomach adenocarcinoma; TGCT: testicular germ cell tumors; THCA: thyroid carcinoma; THYM: thymoma; UCEC: uterine corpus endometrial carcinoma; UCS: uterine carcinosarcoma; UVM: uveal melanoma.

Tanaka et al. found that the levels of *SRPX2 mRNA* in the placenta, lung, trachea, uterus, and adrenal gland were high expression, whereas the levels were relatively low in the peripheral blood, brain, and bone marrow by detecting 24 normal human tissues with real-time RT-PCR [27]. In terms of studies on the understanding of the *SRPX2* gene and its functions in cancer, the mRNA expression from the TCGA and GTEx database (including 29 types of cancer) was analyzed, and *SRPX2* was found to abnormally express in multiple tumors (Figure 2). There is a significant higher expression in several tumor tissues than that of the appropriate normal tissues, including colon adenocarcinoma, lymphoid neoplasm diffuse large B cell lymphoma, glioblastoma multiforme, pancreatic adenocarcinoma, and rectum adenocarcinoma, while there is a significant lower expression in several tumor tissues than that of the appropriate normal tissues, including adrenocortical carcinoma, esophageal carcinoma, kidney chromophobe, kidney renal papillary cell carcinoma, ovarian serous cystadenocarcinoma, prostate adenocarcinoma, thyroid carcinoma, and uterine corpus endometrial carcinoma. Survival analysis indicated that the high expression of *SRPX2* had a shorter overall survival time than that of low expression of *SRPX2.* These findings indicated that abnormal expression of *SRPX2* was closely related to tumor.

## 3. SRPX2 and Inflammation

Idiopathic pulmonary fibrosis (IPF) is a severe lung disease. Its progression is closely related with oxidative stress, mitochondrial dysfunction, and endoplasmic reticulum stress [28]. Fibroblast-to-myofibroblast transition (FMT) has an important role for IPF. Wang et al. used deep-RNA sequencing to analyze the transcriptome between *in vitro* fibroblasts and myofibroblasts. Results showed that SRPX2 was overexpressed, while SRPX2 family other genes, SELE and SELP, were downregulated between *in vitro* fibroblasts and myofibroblasts. In line with *in vitro* results, the expression of SRPX2 in IPF patients' lung was higher than that of control subjects. And it found that TGF-*β*1 mediated the upregulation of SRPX2 *via* the TGF*β*R1/SMAD3/SRPX2/AP1/SMAD7 signaling pathway. Moreover, this study further demonstrated that SRPX2 siRNA-loaded liposomes, administrated by the intratracheal way, displayed good antifibrosis effects on BLM-induced pulmonary fibrosis in mice and significantly reduced FMT [11]. These findings showed that SRPX2 was very important in the progression of pulmonary fibrosis and suggested that targeting SRPX2 might be a good strategy to treat pulmonary fibrosis in clinical setting.

## 4. SRPX2 and Cancer

### 4.1. SRPX2 Involved in Cancer Metastasis, Invasion, and Migration

**Figure 3:**
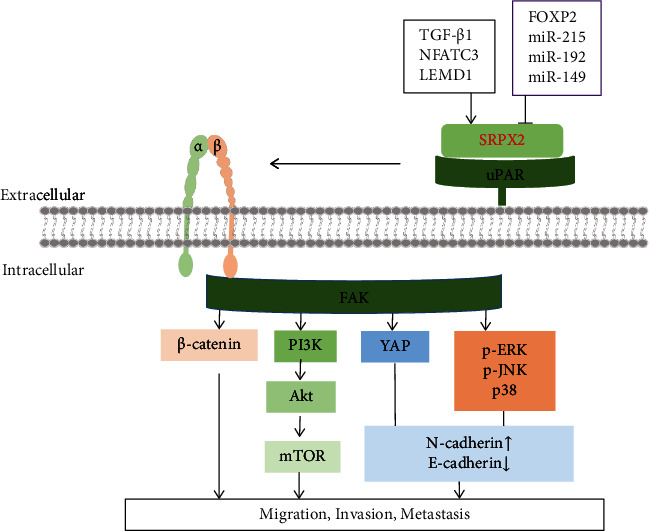
SRPX2 regulates cancer cell metastasis, migration, and invasion by regulating various signaling pathways.

This process of cancer spread is known as metastasis. A major process of metastasis is the proteolytic degradation of the extracellular matrix (ECM) to promote tumor cell migration and invasion [29]. Currently, metastatic and invasive cancers remain largely incurable. The abnormal expression of SRPX2 is a common molecular characteristic of human cancers and enhanced cell metastasis and invasion by regulating the focal adhesion kinase (FAK) signaling pathway, epithelial-mesenchymal transition (EMT), angiogenesis, and glycosylation (Figure 3).

#### 4.1.1. Regulation of the Focal Adhesion Kinase (FAK) Signaling Pathway

FAK is a nonreceptor tyrosine kinase and aggregates to the cytoplasmic tails of integrins to regulate cellular adhesion, migration, invasion, and metastasis by activating a series of cellular signaling pathways such as the matrix metalloproteinases (MMPs), PI3K/Akt, and Wnt/*β*-catenin pathway [30–33]. Researchers showed that elevated SRPX2 in gastric cancer and pancreatic ductal adenocarcinoma enhanced migration and adhesion through increasing phosphorylation levels of FAK [27, 34]. In hepatocellular carcinoma, SRPX2 knockdown suppressed pulmonary metastasis of hepatocellular carcinoma HCCLM3 cells in nude mice, which the mechanisms were through inhibiting FAK/AKT pathway-mediated MMP2/9 expression [23]. Moreover, the inhibition of SRPX2 by siRNA also inhibited the invasion of colorectal cancer HCT116 cells through downregulating Wnt/*β*-catenin pathway-mediated MMP2/9 expression [16]. SRPX2 has been demonstrated to be a ligand for uPAR, and uPAR-integrin interaction is frequently associated with the activation of FAK [35]. Therefore, the uPAR/integrins/FAK/MMPs signaling pathway may play an central role in SRPX2-induced tumor metastasis and invasion.

#### 4.1.2. Regulation of Epithelial-Mesenchymal Transition (EMT)

EMT is a highly dynamic process characterized by transformation of polarized epithelial cells into highly invasive mesenchymal cells, resulting in the increased capacity of migration and invasion [36]. The events occurring during EMT include the loss of adherent junctions, the downregulation of E-cadherin, and upregulation of N-cadherin, Twist, Snail, Slug, fibronectin, MMP-2, and MMP-9 [37]. Wu et al. found that SRPX2 knockdown inhibited osteosarcoma invasion by decreasing N-cadherin levels and increasing E-cadherin level *in vivo*. Further study showed SRPX2 knockdown increased the phosphorylation of Yes-associated protein (YAP), thus decreasing nuclear translocation to activate the Hippo signaling pathway, suggesting that SRPX2 might activate the Hippo signaling pathway to increase cell invasion in osteosarcoma [38]. It was also observed that SRPX2 knockdown inhibited migration and invasion by reducing N-cadherin level and increasing E-cadherin level in prostate cancer cells (LNCaP cells and DU-145cells). Furthermore, SRPX2 knockdown obviously inhibited the phosphorylation of PI3K, Akt, and mTOR, and the treatment with PI3K inhibitor LY294002 efficiently enhanced the inhibitory effects of sh-SRPX2 on LNCap cell migration, suggesting SRPX2 regulates the migration and invasion *via* the EMT process by mediating the PI3K/Akt/mTOR signaling pathway [7]. In glioblastoma, SRPX2 knockdown significantly decreased the expression of EMT markers (N-cadherin, fibronectin, Twist, Snail, and Slug) and increased E-cadherin expression, while SRPX2 overexpression increased N-cadherin and fibronectin expression and decreased E-cadherin expression. In the meantime, it was observed that knockdown of SRPX2 decreased the MAPK signaling pathway (p-ERK, p-JNK, and p-p38) proteins, but blocking the MAPK signaling pathway by PD098059 inhibited glioblastoma metastasis, not cell invasion and migration in the cells with downregulation of SRPX2, suggesting that SRPX2 promoted the EMT process to enhance glioblastoma metastasis through the MAPK signaling pathway [18]. In addition, knockdown of SRPX2 inhibited metastasis in esophageal squamous cell carcinoma cells by preventing the EMT process via the inactivation of the Wnt/*β*-catenin signaling pathway [39]. Therefore, EMT process plays an important role for SRPX2 to induce cancer invasion and metastasis by various pathways including the Hippo signaling pathway, PI3K/Akt/mTOR signaling pathway, MAPK signaling pathway, and Wnt/*β*-catenin signaling pathway.

#### 4.1.3. Regulation of Angiogenesis

Angiogenesis is the process through which novel blood vessels are formed from the existing blood vessels, and provides sufficient supply of nutrients and oxygen to cells. Tumor angiogenesis is closely related with tumor growth, progression, and metastasis [40]. Miljkovic-Licina et al. used in situ mRNA hybridization and immunostaining to detect SRPX2 expression in de novo formation of the blood vessels in angiogenic tissues and found SRPX2-specific expression in tumor endothelial sprouts. This study further demonstrated that silencing of SRPX2 delayed angiogenic sprout formation and SRPX2 bound to vascular uPAR by pull-down experiments [41]. Because SRPX2 is a secretory proteoglycan in extracellular matrix, other study used SRPX2 recombinant protein to further demonstrate that SRPX2 could significantly increase the angiogenic ability of human umbilical vein endothelial cells (HUVECs) through the uPAR/integrin/FAK pathway, which activated the PI3K/Akt and Ras/MAPK pathways [42].

#### 4.1.4. Regulation of Glycosylation

Glycosylation is the most ubiquitous molecular modification, which regulates many biological functions [43]. Compared with *O*-glycans in healthy tissues, cancer-associated *O*-glycans are often truncated [44, 45]. Abnormal glycosylation in cancer is associated with cancer progression and metastasis [46]. MKN45 SC cells and AGS SC cells are derived from two gastric cancer cell lines, MKN45 and AGS, by knocking out the *COSMC* gene, which is essential for *O*-glycan elongation [47, 48]. Freitas and coworkers found that truncation of *O*-glycans in MKN45 SC cells and AGS SC cells promoted invasive features. The results of the transcriptomic analysis exhibited that *COSMC* knockout in MKN45 SC cells and AGS SC cells resulted in a significant alteration in the transcription profile in comparison with the respective parental cells, especially the most upregulated *SRPX2* gene, suggesting that SRPX2 may promote cancer invasion through regulating cell glycosylation [49].

### 4.2. SRPX2 Involved in Cancer Chemosensitivity

Cancer resistance is still the greatest challenge in improving the outcomes for cancer patients [50]. Accumulating evidence demonstrated that SRPX2 was involved in chemosensitivity. Platinum-based chemotherapeutic drugs have been used extensively to treat various human cancers such as esophageal cancer, head and neck cancer, and lung cancer [51]. It has shown that downregulation of SRPX2 restored the sensitivity of oral squamous cell carcinoma cells to nedaplatin and cisplatin and increased the sensitivity of esophageal squamous cell carcinoma cells towards cisplatin [14, 39]. The study by Tang et al. showed that overexpression of SRPX2 induced temozolomide resistance in glioblastoma [18]. Additionally, it was observed that SRPX2 level of pancreatic cancer patients with stable disease and progressive disease after chemotherapy was above that before chemotherapy, while SRPX2 level of pancreatic cancer patients with partial remission after chemotherapy was lower than that before chemotherapy, suggesting that SRPX2 was closely associated with chemotherapeutic efficacy of pancreatic cancer patients. Further study indicated that silencing of SRPX2 sensitized pancreatic cancer cells against 5-Fu and gemcitabine by deactivating the PI3K/AKT/mTOR signaling pathway [52].

### 4.3. SRPX2 as a Prognosic Marker in Cancer

Researchers have found SRPX2 may yield several clinically relevant insights in patients with cancers. Li and coworkers demonstrated that patients with elevated SRPX2 expression are related to shorter disease-free survival (DFS) and overall survival (OS) than patients with low SRPX2 expression by detecting 200 tissue microarray specimens from patients (79 training and 121 validation) with curative pancreatectomy for pancreatic ductal adenocarcinoma [17]. Furthermore, elevated SRPX2 expression was significantly associated with shorter OS in prostate cancer [53]. In patients with papillary thyroid cancer, high SRPX2 expression was demonstrated to as an independent prognostic factor [54]. Moreover, it was also reported that gastric cancer patients with overexpression of SRPX2 presented a lower OS and worse prognosis in comparison to patients with low SPRX2, and SRPX2 was considered a useful independent predictor of outcomes [15]. However, other study detected 112 gastric cancer patients' peripheral blood karyocytes and did not find SRPX2 was related with prognosis [55]. Therefore, SRPX2 may be a prognostic biomarker in tumor tissue, but it needed to further explore the role in peripheral blood karyocytes.

## 5. Conclusion and Perspective

Since its discovery in 1999, SRPX2 is demonstrated to associate with the progression of various types of cancers. Research interest in cancer mainly focused on cancer metastasis, invasion, migration, and drug resistance through regulating the FAK signaling pathway, EMT, angiogenesis, and glycosylation (Table 1). Moreover, SRPX2 was a prognostic factor with OS and DFS through analyzing the tumor tissue, but not in peripheral blood karyocytes (Table 1). Furthermore, SRPX2 exhibited excellent antifibrosis effect *via* the TGF*β*R1/SMAD3/SRPX2/AP1/SMAD7 pathway *in vitro* and *in vivo*. Thus, these findings indicated that SRPX2 might be a potential therapeutic target for treatment of highly metastatic cancers, cancer resistance, and inflammation. Inflammatory signaling pathways are closely involved in tumorigenesis, therapy resistance, and metastasis. A better understanding of the roles of SRPX2 in inflammation will be helpful to treat cancer-related inflammation to slow the progression of cancer. Additionally, it is a pity that there is little connection between SRPX2 and drug development at current. Therefore, in-depth understanding of structure and functions of SRPX2 will provide the theoretical foundations to drug design.

## Figures and Tables

**Table 1:** Roles for SRPX2 in cancer metastasis, invasion, migration, drug resistance, and prognosis.

Cancer type	Downstream signaling pathway	Upstream signaling pathway	Effects	Prognostic value	References
Gastric cancer	FAK	/	Migration and adhesion induction	Lower OS and worse prognosis; independent predictor of outcomes	[15, 27]
Colorectal cancer	Wnt/*β*-catenin pathway-mediated MMP2/9	miR-192, miR-215, miR-149	Invasion induction	/	[16]
Pancreatic ductal adenocarcinoma	FAK; PI3K/AKT/mTOR	/	Migration and adhesion induction; 5-Fu and gemcitabine resistance	Shorter DFS and OS	[17, 34, 52]
Prostate cancer	PI3K/Akt/mTOR	/	Migration and adhesion induction	Shorter OS	[7, 53]
Oral squamous cell carcinomas	/	LEMD1	Adhesion induction; nedaplatin and cisplatin resistance	/	[10]
Glioblastoma	p-ERK, p-JNKp38	/	Metastasis induction; temozolomide resistance	/	[18]
Hepatocellular carcinoma	FAK/AKT pathway-mediated MMP2/9	/	Metastasis induction	/	[19]
Osteosarcoma	Yes-associated protein (YAP)	/	Invasion induction	/	[38]
Esophageal squamous cell carcinoma	Wnt/*β*-catenin	/	Metastasis induction; cisplatin resistance	/	[39]
Papillary thyroid cancer	/	/	/	Independent prognostic factor	[54]

## Data Availability

The data supporting this review are from previously reported studies and datasets, which have been cited. The processed data generated or analyzed during this study are included in this article.
